# ‘Two-level’ measurements of processing speed as cognitive markers in the differential diagnosis of DSM-5 mild neurocognitive disorders (NCD)

**DOI:** 10.1038/s41598-017-00624-8

**Published:** 2017-03-31

**Authors:** Hanna Lu, Sandra S. M. Chan, Linda C. W. Lam

**Affiliations:** 1 Guangzhou Brain Hospital, The Affiliated Brain Hospital of Guangzhou Medical University, Guangzhou, China; 2 Department of Psychiatry, The Chinese University of Hong Kong, Hong Kong SAR, China

## Abstract

Processing speed is an updated diagnostic factor for neurocognitive disorders (NCD) in DSM-5. This study investigated the characteristics of processing speed and their diagnostic values in NCD patients. A flanker test was conducted in 31 adults with NCD due to vascular disease (NCD-vascular), 36 patients with NCD due to Alzheimer’s disease (NCD-AD), and 137 healthy controls. The processing speed was evaluated using two measurements: mean reaction time (RT) and intra-individual variability of RT. Mean RT represents the global processing speed. Intra-individual variability of RT is the short-term fluctuation of RT and consists of two indices, which are intra-individual coefficient of variation of reaction time (ICV-RT) and intra-individual standard deviations (iSD). We observed elevated ICV-RT and iSD in NCD-AD and NCD-vascular patients. Additionally, there was a slowed RT in NCD-AD patients. The intra-individual variability of RT had a moderate power to differentiate NCD subgroups. The mean RT was able to discriminate the NCD-AD from NCD-vascular patients. Our findings highlight the clinical utility of the combined ‘two-level’ measurements of processing speed to distinguish between individuals with different cognitive status. Furthermore, the ‘two-level’ features of processing speed embedded in the psychometric property may also reflect the diverse aetiology underlying certain ‘disease-specific’ neurocognitive disorders.

## Introduction

In the current conceptual framework of dementia disease trajectory, the neurocognitive disorders (NCD) are a new term that replaces ‘mild cognitive impairment’ (MCI) and represents an intermediate state prior to clinical dementia^1^. Processing speed now serves as one of the key diagnostic items within the new diagnostic framework defined in the Diagnosis and Statistical Manual 5^th^ edition (DSM-5)^2^. The updated inclusion of NCD as part of the DSM-5 diagnostic criteria highlights the importance of understanding and characterizing processing speed in the diagnostic evaluation of NCD.

Processing speed is a well-known indicator of brain efficacy and has been extensively used to study MCI and dementia^3–5^. A slowed reaction time (RT) is often accompanied by multiple errors and is one of the most common manifestations of impaired processing speed^3^. However, processing speed is not a simple static measure of RT and is a composite dynamic measure that accounts for two sources of variability. The sources include inter-individual variability due to diagnostic classification, while NCD refers to a community with diverse aetiology, neurocognitive profile, and clinical outcomes^6, 7^. The two major subtypes of NCD described in the DSM-5 are due to vascular disease (NCD-vascular) or Alzheimer’s disease (NCD-AD)^2^. NCD-vascular and NCD-AD patients have shown group-wise differences with respect to mean RT^8^. There is also intra-individual variability due to advancing age, and this variability reflects the short-term fluctuations on cognitive tests. This type of variability is a universal feature that progressively increases during the ageing process^9, 10^. Thus, elevated intra-individual variability has been found in individuals with MCI^11–13^, amnestic MCI^14^, Alzheimer’s disease^15^ and mild dementia^16^ relative to age-matched healthy controls. The increased intra-individual variability of RT is an independent marker that can predict the conversion from the pre-clinical stage to clinical dementia^17, 18^.

Cumulatively, the mean RT and intra-individual variability of RT represent the ‘two-level’ measurements of processing speed. It is currently unclear whether the two measurements of processing speed can be used to differentiate senior adults with different cognitive status independently or in combination. Thus, the primary objective of the current study was to investigate the clinical utility of ‘two-level’ measurements of RT in discriminating subtypes of NCD using the following approaches: (*i*) measuring mean RT and intra-individual variability of RT in NCD patients and healthy counterparts; (*ii*) classifying subtypes of NCD using the two measures of processing speed. As a secondary objective, we explored the correlations between mean RT, intra-individual variability of RT, and scores on neurocognitive tests.

## Results

### Demographics and neurocognitive performance

The basic participant demographics, including gender, age, and years of education, were similar for the three groups examined. The NCD subgroups showed a decline of global cognition (Table 1). The NCD-AD group had the worst performance in the domain of short-term memory (measured by delayed recall). The NCD-vascular group exhibited marked and extensive cardiovascular burden with the presence of higher scores on the cumulative illness rating scale (CIRS) (Cardiovascular risk factor: *F* = 3.28, *p* = 0.04; Heart disease: *F* = 9.11, *p* < 0.001; Lipid: *F* = 4.395, *p* = 0.014). The NCD-vascular group also presented poorer executive function (measured by Chinese Verbal Fluency Test, CVFT) than the NCD-AD group.

**Table 1:** Demographics and neurocognitive performance between healthy and NCD groups.

	Healthy (n = 137)	NCD-AD (n = 36)	NCD-Vascular (n = 31)	*F* (*χ* ^*2*^)	*P value*
Age	71.45 ± 3.99	73.35 ± 4.91	73.46 ± 5.27	3.098	0.056
Gender (F/M)	61/76	16/20	12/19	1.800	0.168
Education (years)	9.30 ± 4.26	7.56 ± 3.92	8.21 ± 3.27	3.141	0.055
CSDD	0.49 ± 1.81	0.18 ± 0.72	0.26 ± 0.85	0.590	0.555
PSQI	5.75 ± 3.15	6.24 ± 3.69	5.88 ± 3.05	1.284	0.279
ADL	6.09 ± 3.37	5.19 ± 3.12	6.35 ± 3.27	3.139	0.061
CDR-SOB	0.34 ± 0.47	0.81 ± 0.54	0.81 ± 0.70	17.915	<0.001
CMMSE	28.63 ± 1.17	27.22 ± 1.96	26.74 ± 1.73	30.869	<0.001
HK MoCA	27.23 ± 1.85	24.11 ± 3.21	24.00 ± 2.77	44.020	<0.001
ADAS-Cog	4.91 ± 2.08	9.42 ± 2.49	8.98 ± 2.63	82.615	<0.001
Delayed recall	7.64 ± 1.45	3.97 ± 0.91	5.97 ± 1.08	115.717	<0.001
Digit span backward (DSB)	3.75 ± 1.32	3.00 ± 1.22	3.03 ± 0.84	7.863	0.001
CVFT	47.71 ± 9.18	39.81 ± 8.26	35.29 ± 5.96	32.511	<0.001
Trail making test B	67.07 ± 41.26	91.21 ± 62.26	83.17 ± 38.13	4.784	0.009
Trail making test A	12.90 ± 6.37	16.87 ± 10.18	15.62 ± 6.09	5.327	0.006
Digit span forward (DSF)	7.62 ± 1.13	7.17 ± 0.94	7.00 ± 1.44	5.118	0.007

Note. Data are raw scores and presented as mean ± SD. CSDD = The Cornell Scale for Depression in Dementia; PSQI = Pittsburgh Sleep Quality Index; ADL = Activity of daily living scale; CDR-SOB = Clinical dementia rating-sum of box; CVFT = Chinese verbal fluency test.

### ‘Two-level’ measurements of processing speed

**Figure 1:**
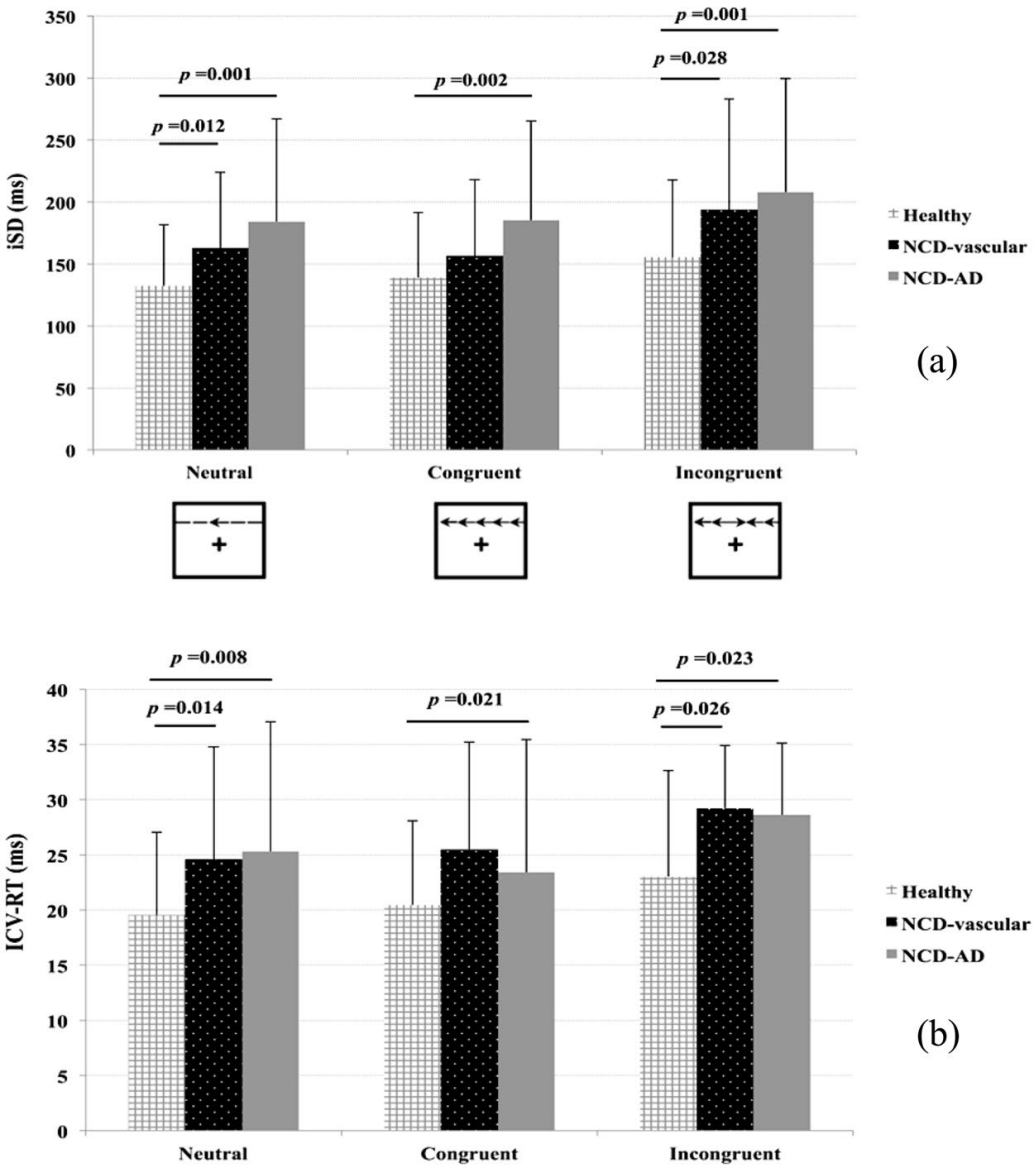
Intra-individual variability of RT between healthy and NCD subgroups. Elevation in iSD (**a**) and ICV-RT (**b**) has been found in NCD-AD and NCD-vascular patients.

The data show there were prominent inter-individual differences in mean RT using conditions with three types of flanker tests (Table 2). The NCD-AD group showed marked slowing in RT compared to healthy controls (HC) (mean RT: *t* = 2.662, *p* = 0.011) and the NCD-vascular group (mean RT: *t* = 2.318, *p* = 0.024). The NCD-vascular group showed similar mean RT as the HC. The second level of processing speed showed elevated short-term fluctuations on RT performance in both the NCD-AD and NCD-vascular groups (Table 3). The NCD subgroups showed greater iSD (Fig. 1a) and ICV-RT across the conditions with three flanker types (Fig. 1b).

**Table 2:** Comparisons of mean RT between healthy and NCD subgroups.

	Healthy (n = 137)	NCD-AD (n = 36)	NCD-Vascular (n = 31)	*F*	*P* value
RT of Neutral	652.69 ± 105.78	707.96 ± 120.81	644.34 ± 91.82	4.284	0.015
RT of Congruent	666.39 ± 109.80	715.00 ± 135.85	653.42 ± 92.29	3.218	0.042
RT of Incongruent	726.25 ± 107.88	811.96 ± 143.06	742.29 ± 108.84	7.930	<0.001
Mean RT	687.49 ± 105.97	750.59 ± 132.59	685.32 ± 97.09	4.958	0.008

**Table 3:** Indices of intra-individual variability of RT between healthy and NCD subgroups.

	Healthy (n = 137)	NCD-AD (n = 36)	NCD-Vascular (n = 31)	*F*	*P* value
**iSD**					
Neutral	132.39 ± 49.48	184.26 ± 82.55	163.17 ± 60.64	12.910	<0.001
Congruent	138.98 ± 52.40	185.20 ± 80.30	156.58 ± 61.54	8.796	<0.001
Incongruent	155.50 ± 62.36	208.04 ± 91.60	193.98 ± 88.88	9.361	<0.001
Average iSD	142.29 ± 49.71	192.50 ± 79.92	171.25 ± 65.58	11.803	<0.001
**ICV-RT**					
Neutral	19.54 ± 7.52	25.31 ± 11.77	24.56 ± 10.21	8.536	<0.001
Congruent	20.43 ± 7.64	25.50 ± 12.05	23.41 ± 9.73	5.271	0.006
Incongruent	23.01 ± 9.63	28.63 ± 13.42	29.23 ± 14.25	6.325	0.002
Average ICV-RT	19.99 ± 4.57	23.80 ± 6.52	22.35 ± 5.70	9.07	<0.001

### Receiving operating characteristic (ROC) analysis

**Figure 2:**
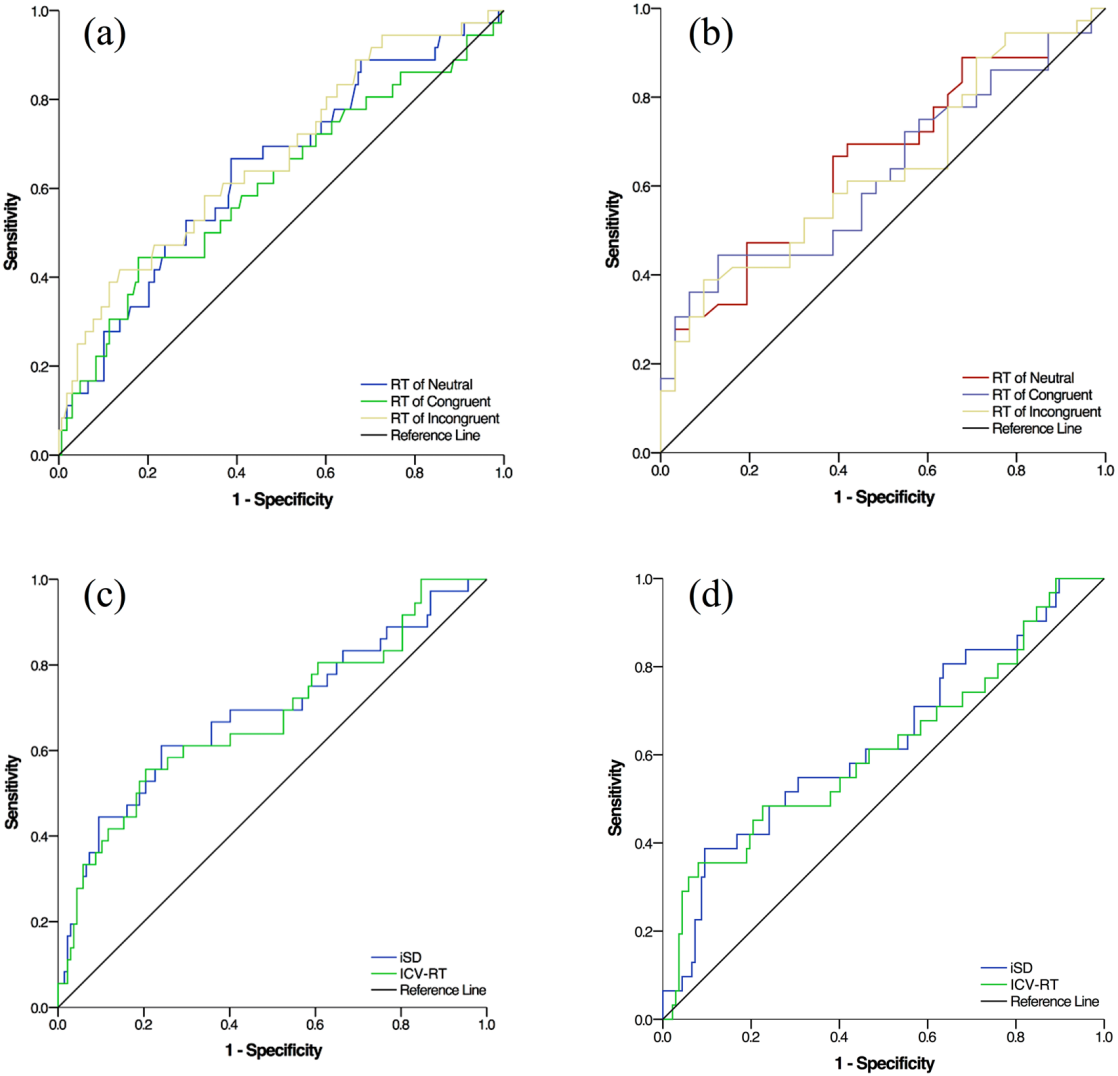
Receiver operator characteristic curves for the ‘two-level’ measurements of processing speed in the adults with different cognitive status. Mean RT of three flanker types presents a modest power to differentiate NCD-AD (**a**) from healthy and NCD-vascular groups (**b**). The indices of intra-individual variability of RT shows a utility to discriminate NCD-AD (**c**) and NCD-vascular (**d**) from healthy counterparts.

The data in Fig. 2a show that the area under the curve (AUC) value of mean RT could distinguish the NCD-AD from HC (RT of neutral = 0.642, *p* = 0.007; RT of congruent = 0.611, *p* = 0.037; RT of incongruent = 0.672, *p* = 0.001). Furthermore, the mean RT (Fig. 2b) also demonstrated a modest power to differentiate the NCD-AD from NCD-vascular patients (AUC value = 0.655, *p* = 0.03). It is interesting to note that the mean RT under the condition with higher levels of cognitive demand (i.e., incongruent) had higher discriminatory power in differentiating NCD-AD patients from HC. Conversely, the mean RT under the condition with lower levels of cognitive demand (i.e., neutral) had higher discriminatory power in differentiating between NCD-AD from NCD-vascular patients.

The second level measurement of processing speed, including iSD (AUC value = 0.687, *p* = 0.001) and ICV-RT (AUC value = 0.677, *p* = 0.001), was able to distinguish NCD-AD cases from HC (Fig. 2c). Similarly, the measure of intra-individual variability also demonstrated a modest power to discriminate NCD-vascular cases from HC (iSD: AUC value = 0.631, *p* = 0.023; ICV-RT: AUC value = 0.615, *p* = 0.045) (Fig. 2d). However, neither iSD nor ICV-RT could distinguish between individuals with subtypes of NCD.

### Associations between RT measures and neurocognitive function

**Figure 3:**
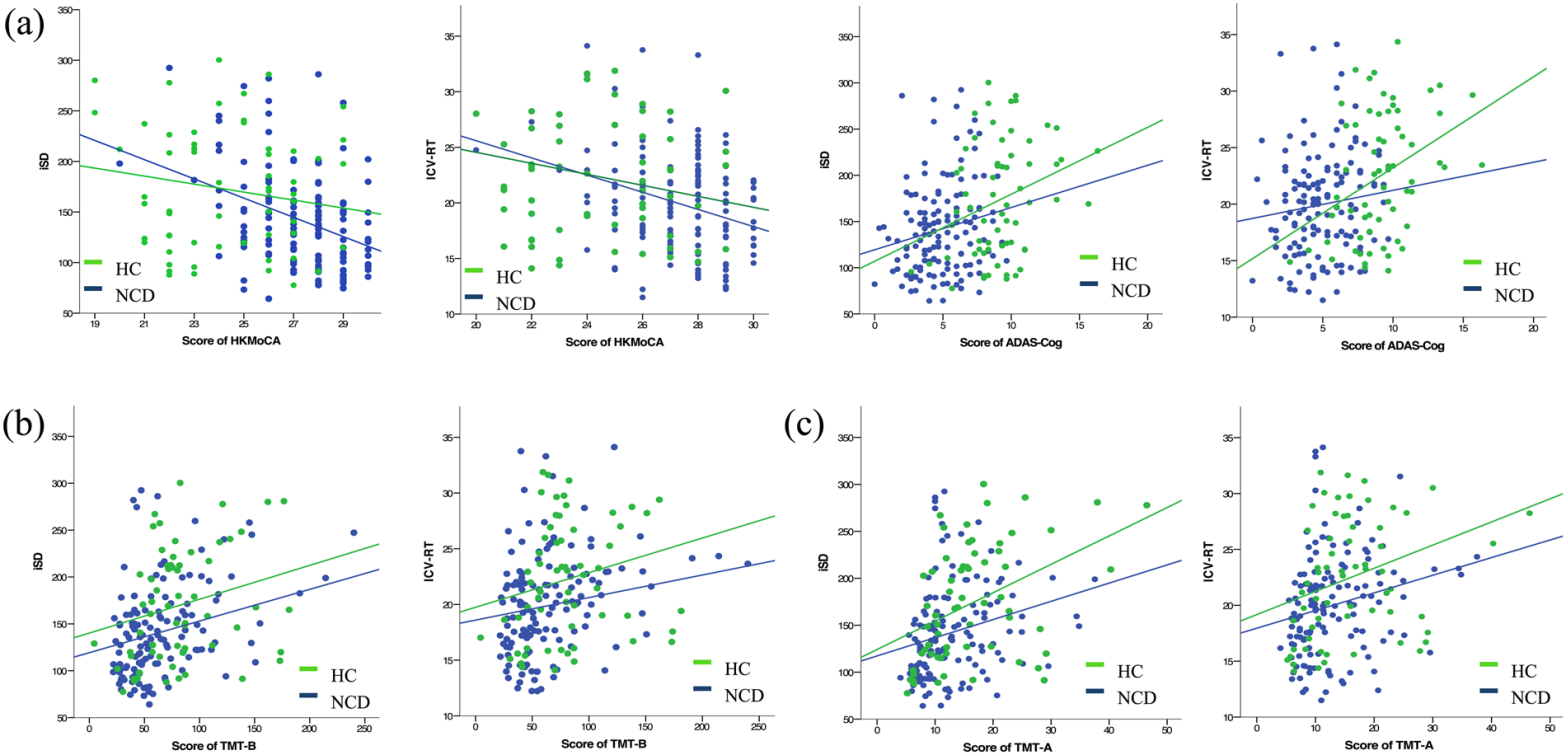
Correlations between RT measures and neurocognitive function. Elevated intra-individual variability of RT was associated with the worse performance of global cognition (**a**), executive function (**b**), and attention (**c**).

The data indicate that covariates, such as age, gender, and years of education, increased intra-individual variability of RT. These covariates were extensively correlated with worse neurocognitive performance in the healthy and NCD groups and were associated with the following tests of global cognition (Fig. 3a): HK MoCA (HC: iSD: *r* = −0.352, *p* < 0.001; ICV-RT: *r* = −0.31, *p* < 0.001; NCD: ICV-RT: *r* = −0.274, *p* = 0.028), ADAS-Cog (HC: iSD: *r = *0.192, *p* = 0.025; NCD: iSD: *r* = 0.293, *p* = 0.019; ICV-RT: *r = *0.368, *p* < 0.001), executive function evaluated by TMT-B (Fig. 3b, HC: iSD: *r = *0.281, *p* = 0.001; ICV-RT: *r = *0.183, *p* = 0.033; NCD: iSD: *r = *0.314, *p* = 0.012; ICV-RT: *r = *0.301, *p* = 0.017), and attention (Fig. 3c) assessed by TMT-A (HC: iSD: *r = *0.248, *p* = 0.003; ICV-RT: *r = *0.22, *p* = 0.01; NCD: iSD: *r = *0.421, *p* = 0.001; ICV-RT: *r = *0.327, *p* = 0.008).

## Discussion

This is the first study to examine the ‘two-level’ measurements of processing speed and their discrimination properties in senior adults with NCD-AD and NCD-vascular. Our findings are consistent with previously reported empirical evidence^9, 10, 13–15, 17^ and showed there was slowed mean RT and elevated intra-individual variability in disease-specific NCD, despite having limited utility in differentiating between subtypes. Additionally, a combination of the two measures could differentiate between patients with different cognitive status.

Emerging evidence has shown these measures can distinguish between individuals with various cognitive deficits in preclinical dementia^14, 19^ and early dementia^20, 21^. However, it is important to note that cognitive performance characterized by different tests with different scoring methods across studies could lead to heterogeneous results. The ‘two-level’ measures of processing speed derived from the same test (i.e., Flanker test) have shown the ability to discriminate the subtypes of NCD, which is an emerging need in clinical differential diagnosis^22, 23^.

The slowed processing speed among senior adults may result from reduced ability to inhibit competing and irrelevant responses when resolving the conflict^24, 25^. Our results differed from previous published data, and we found a markedly slowed mean RT in NCD-AD but a comparable mean RT in NCD-vascular cases^18, 26^. Our observations may be partly explained by the different trajectories of neurocognitive change between subtypes of NCD. For example, in contrast to the thorough breakdowns in NCD-AD cases, NCD-vascular patients show a diverse pattern of disturbed processing speed. The compensatory strategies might allow the senior adults with NCD-vascular to maintain a level of mean RT similar to healthy controls at the early stage of preclinical dementia. Alternatively, the ability to preserve consistent performance of RT (i.e., intra-individual variability of RT) has already been reduced. The elevated intra-individual variability of RT parallels pathological ageing. Due to the coupled changes between ageing and brain efficacy^27^, a tempting speculation is that the intra-individual variability is an elementary measure engaged broadly in neurocognitive function.

The short-term fluctuations in RT were associated with the scores on multiple neurocognitive tests (Fig. 3). The results suggest that intra-individual RT variability might serve as a fundamental neurocognitive marker of brain function. In addition, the linkages between intra-individual variability of RT and neurocognitive performance have also exemplified the ontogenetic changes that occur during the process of pathological ageing. The robust “intra-individual variability-cognition” correlation provides preliminary support for the use of variability indices as a neurocognitive proxy marker in conventional neuropsychological tests. Although there are several cognitive tests (i.e., trail making test) commonly employed in diagnosing preclinical dementia^28, 29^, it is generally difficult to achieve a consensus on the cut-off scores needed for different cognitive tests^30^. Moreover, there is currently no inclusion of any proven ‘disease-specific’ neurocognitive profile in the diagnostic criteria for NCD in DSM-5^2^. The challenge in collecting neurocognitive features for diagnosing individuals with subtypes of NCD is in a transition stage, and assessment tools with comparable scores across diverse psychometric scales must be developed (i.e., second or millisecond).

In conclusion, the inter-individual and intra-individual variability of RT represents the ‘two-level’ measurements of processing speed and may serve as potential neurocognitive phenotypes that discriminate between subtypes of prodromal dementia. The features of processing speed may also provide an interpretable perspective and aid in clarifying the complex relationships between neurocognitive measures.

### Limitations and future work

The results of this study should be interpreted with caution due to several limitations. First, the sample size of each subgroup was unbalanced. We tested the homogeneity of variance before performing the ANOVA to detect the group-wise differences and address this issue. Second, the cross-sectional design had very limited power to infer any causative or ageing influence. Third, the classifications of healthy and NCD subgroups were based on the performance of neurocognitive tests and were not ascertained by neuroimaging or blood biomarker investigations. Additionally, the absence of magnetic resonance imaging (MRI) in the diagnosis of NCD-vascular group patients was a major limitation. We conducted detailed CIRS to infer the condition of cerebrovascular burden of each participant. Future studies should involve structural MRI, including T2-weighted MRI, and should perform a cross-validation of the diagnosis in NCD-vascular cases.

## Methods

### Participants

This study recruited 204 community-dwelling adults aged from 65–80 years from another cohort study aiming to establish a detailed characterization of cognitive profiles of Chinese senior adults. A structured neuropsychological battery was conducted to evaluate the global cognition and three major domains of neurocognitive function^31^. The procedures for all studies were approved by the Joint Chinese University of Hong Kong - New territories East Cluster Clinical Research Ethics Committee. Written informed consent was obtained from each participant before the assessment was conducted. All experiments were performed in accordance with the approved guidelines.

### Criteria for selection of healthy and NCD subgroups

(1) Healthy elderly refers to subjects with cognitive performance within 1.5 standard deviation (SD) of age-normal reference derived from the cohort study^32^, which presented with Clinical Dementia Rating (CDR) score equal to 0 and Cantonese Mini Mental State Examination (CMMSE) score greater than 28. (2) The NCD patients are defined by the following three criteria^2^: evidence of modest cognitive decline in one or more cognitive domains, which was set as ≥1.5 SD below the cognitive performance of healthy elderly; no interference with independence in everyday activities; and no better explanation by other psychiatric disorders. NCD-vascular and NCD-AD patients fulfilled the criteria of NCD. The definition of NCD-vascular cases required more than two chronic cerebrovascular risks and reduced functioning across the cognitive domains except for memory. NCD-AD cases demonstrated declined functioning in memory. (3) The exclusion criteria for this study included the following: clinical dementia, defined as cases with CMMSE score below the local cut off for dementia of 18 and below for illiterate elderly, 20 and below for those with one to two years of education, and 22 and below for subjects with more than two years of education^33^; and cases with depressive symptoms, sleep disorders, or history of neurological or psychiatric disorders.

### Neurocognitive assessment and clinical evaluation

The Montreal Cognitive Assessment Hong Kong version (HK MoCA), CMMSE, Alzheimer’s Disease Assessment Scale cognitive part (ADAS-Cog), and CDR were used to measure global cognition^34^. The other three major domains of cognitive function included the following^8^: (1) short-term memory measured by delayed recall of words and digit span backward (DSB), (2) attention measured by digit span forward (DSF) and trail making test part A (TMT-A) and (3) executive function measured by trail making test part B (TMT-B) and Chinese verbal fluency test (CVFT). The cerebrovascular risks were evaluated by CIRS^35^ for the presence and severity of hypertension, hyperlipidaemia, heart diseases, diabetes mellitus, atrial fibrillation, and anaemia. The Cornell scale for depression in dementia (CSDD)^36^, Pittsburgh sleep quality index (PSQI)^37^, and activity of daily living scale (ADL) were used to assess the depression symptoms^38^, sleep disorders, and everyday functioning separately. All the measurements were conducted with Chinese instructions.

### Mean RT and intra-individual variability of RT

The intra-individual variability refers to the ‘trial-to-trial’ fluctuations on cognitive performance through repeated measurements. The flanker test (with arrows)^39^ is a computer-based test that contains 288 trials for collecting the RT and is suitable for measuring the intra-individual variability of RT. In a given trial of the flanker test, a cross-fixation point presents for 400 to 1600 millisecond (ms) (randomized) and is subsequently replaced for 100 ms by the warning cues. The target is a central arrow that appears above or below the cross-fixation and is surrounded by two flankers on each side. The tree types of flanker include neutral (− − > − −), congruent (< < < < <) and incongruent (< < > < <) and implicate the different levels of cognitive demand. All participants were instructed to decide whether a central arrow points to left or right before the test. The subjects pressed the left button of the mouse if the central arrow was pointing to left and the right button if it was pointing to right. All participants were instructed to respond as rapidly as possible to the direction of the flanker by clicking the left or right button. The reaction time was the completion time in milliseconds for a given trial and was used to calculate the mean RT and intra-individual variability of RT.

We calculated two indices of intra-individual variability to more thoroughly detect the intra-individual variability of RT and the possible effects of mean RT on intra-individual variability. The first index was the intra-individual standard deviation (iSD), which was computed across the trials of flanker test with the presence of mean SD of RT. We followed the suggestion from Hultsch *et al*.^40^ regarding chronological age and categorical trials, and their interactions were regressed out to minimize the potential confounding influence. The residuals were converted to the standardized scores (z-scores) and used as the measure of iSD. Due to the slowed processing speed in old age, we determined the intra-individual coefficient of variation of RT (ICV-RT) to assess the intra-individual variability using the following formula: ICV-RT = (SD of RT/mean RT) × 100^41, 42^. Higher values of iSD and ICV-RT indicate elevations in short-term fluctuations on the flanker test.

### Statistical analysis

We performed homogeneity of variance tests to evaluate the equality of variances among the healthy, NCD-vascular, and NCD-AD groups. Group-wise comparisons were tested either with Chi-square test for categorical variables or with one-way analysis of variance (ANOVA) for continuous variables. The Tukey method was used to perform post hoc multiple comparisons as needed. For the flanker test, the median values of RT across the trials were employed as raw scores to avoid the influence of outliers. The ROC analysis was used to evaluate the values of mean RT and intra-individual variability of RT in differentiating the elderly with different cognitive status. The Pearson correlation coefficients were used to detect the relationships between measures of processing speed and neurocognitive features. The mean RT and intra-individual variability of RT were calculated by E-Data Aid embedded in E-Prime 2.0 (Psychology Software Tools, Pittsburgh, PA). The chi-square test, ANOVA, Pearson correlation analysis and ROC analysis were performed by IBM SPSS 20.
